# Conserved and Diverse Transcriptional Reprogramming Triggered by the Establishment of Symbioses in Tomato Roots Forming *Arum*-Type and *Paris*-Type Arbuscular Mycorrhizae

**DOI:** 10.3390/plants11060747

**Published:** 2022-03-11

**Authors:** Takaya Tominaga, Luxi Yao, Hikaru Saito, Hironori Kaminaka

**Affiliations:** 1 The United Graduate School of Agricultural Science, Tottori University, Tottori 680-8553, Japan; d20a1007c@edu.tottori-u.ac.jp; 2 Faculty of Agriculture, Tottori University, Tottori 680-8553, Japan; jpsmartlucy168@gmail.com (L.Y.); b18a5077y@edu.tottori-u.ac.jp (H.S.)

**Keywords:** arbuscular mycorrhizal symbiosis, comparative transcriptomics, *Arum*-type, *Paris*-type, *Solanum lycopersicum*, *Rhizophagus irregularis*, *Gigaspora margarita*

## Abstract

Arbuscular mycorrhizal (AM) fungi allocate mineral nutrients to their host plants, and the hosts supply carbohydrates and lipids to the fungal symbionts in return. The morphotypes of intraradical hyphae are primarily determined on the plant side into *Arum*- and *Paris*-type AMs. As an exception, *Solanum lycopersicum* (tomato) forms both types of AMs depending on the fungal species. Previously, we have shown the existence of diverse regulatory mechanisms in *Arum*- and *Paris*-type AM symbioses in response to gibberellin (GA) among different host species. However, due to the design of the study, it remained possible that the use of different plant species influenced the results. Here, we used tomato plants to compare the transcriptional responses during *Arum*- and *Paris*-type AM symbioses in a single plant species. The tomato plants inoculated with *Rhizophagus irregularis* or *Gigaspora margarita* exhibited *Arum*- and *Paris*-type AMs, respectively, and demonstrated similar colonization rates and shoot biomass. Comparative transcriptomics showed shared expression patterns of AM-related genes in tomato roots upon each fungal infection. On the contrary, the defense response and GA biosynthetic process was transcriptionally upregulated during *Paris*-type AM symbiosis. Thus, both shared and different transcriptional reprogramming function in establishing *Arum*- and *Paris*-type AM symbioses in tomato plants.

## 1. Introduction

Approximately 80% of terrestrial plants establish a symbiotic relationship with Glomeromycotina fungi; this relationship is referred to as arbuscular mycorrhizal (AM) symbiosis [1]. Recently, how host plants and AM fungi communicate in the rhizosphere has been elucidated. AM fungal-derived short-chain chitooligosaccharides (COs) and lipo-chitooligosaccharides (LCOs) activate symbiotic signaling in host plants together with some receptor-like kinases (RLKs) [2,3,4,5]. Although AM fungal colonization triggers transient and weak defense responses in the host plants, AM fungi are known to exude some signal components to suppress plant immunity [6,7,8,9]. On the contrary, host plant roots exude strigolactones (SLs) to inform AM fungi of their presence and promote fungal growth [10,11]. SLs are classified as phytohormones and enzymatically biosynthesized from all-trans-β-carotene by DWARF 27 (D27), CAROTENOID CLEAVAGE DIOXYGENASE 7 (CCD7), CCD8, and MORE AXILLARY GROWTH 1 (MAX1) [12,13]. After AM fungi reach the host roots, some AM-specific reprogramming begins in the host root cells. For instance, several studies have demonstrated AM-promoted expression of some symbiotic genes, namely, *PHOSPHATE TRANSPORTER 4* (*PT4*) and *AMMONIUM TRANSPORTER 2;3* (*AMT2;3*) for symbiotic phosphate and ammonium transport, *REDUCED ARBUSCULAR MYCORRHIZA 2* (*RAM2*) and G-type ABC transporters (*STR/STR2*) for lipid production and transport, and *VAPYRIN* (*VPY*) for arbuscule development [14,15,16,17,18,19]. These AM-related genes are transcriptionally activated by several transcription factors, such as RAM1 and REQUIRED FOR ARBUSCULE DEVELOPMENT 1 (RAD1), which belong to the GRAS, GIBBERELLIC-ACID INSENSITIVE (GAI), REPRESSOR of GAI (RGA), and SCARECROW (SCR) transcription factor families [20,21,22,23,24]. These are known to localize on the host-derived peri-arbuscular membrane surrounding arbuscules in highly branched hyphal structures formed in root cortical cells [25].

Depending mainly on the host plant species, the mutualism established results in either *Arum*- or *Paris*-type hyphal structures of intraradical AM fungi [26,27,28,29,30]. In *Arum*-type AM, AM fungal hyphae elongate in the apoplastic space of plant cells and form arbuscules in the cortical cells. *Paris*-type AM is characterized by intracellular hyphal elongation and hyphal coils in the host cortical cells. Despite the distinct AM morphologies, the reasons why AM morphotypes vary among host plants has been unclear. In addition, how host plants differentially regulate AM symbioses for each AM morphotype is not fully understood.

To elucidate these enigmas, we have recently compared the regulatory mechanisms underlying *Arum*- and *Paris*-type AM symbioses using several phylogenetically distant host species [31,32]. Interestingly, the phytohormone gibberellin (GA) promotes the establishment of *Paris*-type AM symbiosis in *Eustoma grandiflorum* and *Primula malacoides* [31], whereas GA suppresses *Arum*-type AM symbiosis in legume plants and rice [33,34,35,36]. Notably, one of our previous studies demonstrated that AM fungal colonization transcriptionally promotes conserved symbiotic genes such as *STR* and *RAM1* among three host plants forming distinct AM morphotypes [32]. This previous study also showed that GA treatment alters the transcriptional responses of the symbiotic genes among the examined host plants. These studies lead us to predict that host plants have evolved to change the upstream mechanisms that regulate AM symbioses according to AM morphotype. However, previous comparative analysis on *Arum*- and *Paris*-type AM symbioses cannot thoroughly investigate our hypothesis due to the possibility that the use of different plant species influenced the results. For a more precise comparison of *Arum*- and *Paris*-type AM symbiosis regulation, a single host plant that forms both types of AMs is needed to overcome these issues. 

Interestingly, *Solanum lycopersicum* (tomato) mainly has *Arum*-type AM in response to Glomeraceae fungi, such as *Rhizophagus irregularis* (formerly *Glomus intraradices*) and *Paris*-type AM, in response to Gigasporaceae fungi, such as *Gigaspora margarita* [6,37]. Here, we conducted comparative analyses on *Arum*- and *Paris*-type AM symbioses in tomato roots colonized by *R. irregularis* and *G. margarita*. *R. irregularis* and *G. margarita* infection showed *Arum*- and *Paris*-type AMs in tomato roots, respectively, and significantly promoted tomato shoot growth. In addition, transcriptome profiling of the tomato roots showed that colonization by either AM fungus triggered some shared transcriptional reprogramming in AM-related genes and the SL biosynthetic pathway in tomato roots. On the contrary, the immune response and the GA biosynthetic process were transcriptionally upregulated in *Paris*-type AM symbiosis in tomato roots. Therefore, our comparative transcriptomics identified both shared and different reprogramming in a single host species during *Arum*- and *Paris*-type AM symbioses.

## 2. Results

### 2.1. Comparison of Symbiotic Phenotypes in Tomato Plants Forming Distinct AM Morphotypes

**Figure 1:**
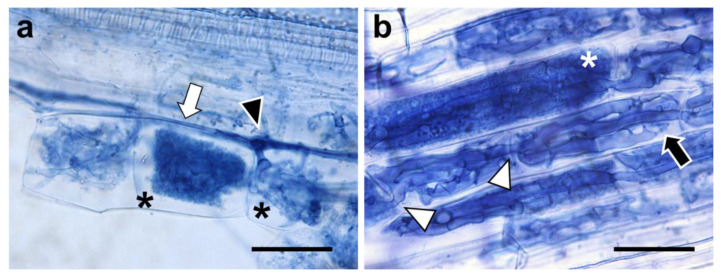
Arbuscular mycorrhizal (AM) morphotypes formed in tomato roots colonized by *Rhizophagus irregularis* or *Gigaspora margarita*. Tomato roots inoculated with *R. irregularis* (**a**) or *G. margarita* (**b**) were collected five weeks post-inoculation (wpi). The collected root samples were stained with 0.05% trypan blue. Scales: 20 µm. White arrow, intercellular hypha; black arrowhead, intercellular hypha penetrating the tomato cortical cell; asterisks, arbuscules; black arrow, hyphal coil; white arrowheads, intracellular hypha penetrating the adjacent tomato cortical cells.

Tomato mycorrhizal roots were sectioned and then subjected to microscopical observation of the AM fungal morphotypes of *R. irregularis* and *G. margarita* in tomato roots. Tomato roots colonized by *R. irregularis* had fungal hyphae in the intercellular space and arbuscules emerged from the intercellular hypha in the cortical cells (Figure 1a) in a manner typical of *Arum*-type AM. On the contrary, *G. margarita* infection exhibited classic *Paris*-type AM with a thick and clear hyphal coil to which the arbuscule adhered and intracellular hyphal elongation in the tomato root cortex (Figure 1b). As several reports have demonstrated [37,38,39], we confirmed that AM fungal traits determine the AM morphotypes of tomato plants under the same growth conditions.

**Figure 2:**
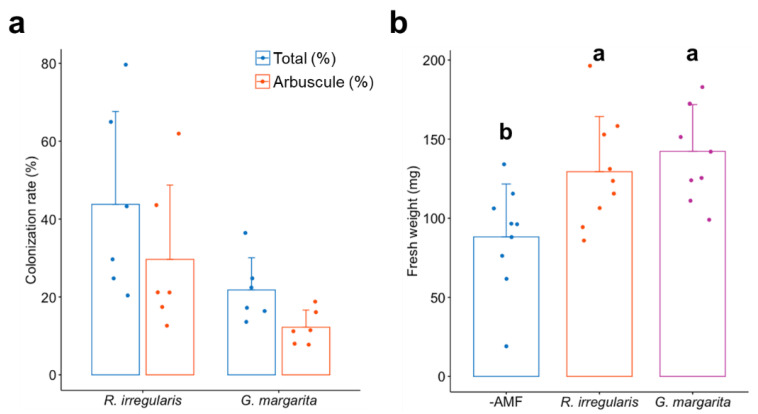
Quantification of AM fungal colonization rates and AM-promoted shoot growth of tomato plants. (**a**) The fungal colonization rates of *Rhizophagus irregularis* or *Gigaspora margarita* in tomato roots at five weeks post-inoculation (wpi). Total (%), the percentage of all hyphal structures observed in the tomato roots; arbuscule (%), the rate of arbuscules formed in the cortical cells. There is no statistically significant difference in the colonization rates between *R. irregularis* and *G. margarita* in Welch’s *t*-test. The error bars show the standard errors (n = 6). (**b**) The shoot fresh weight of tomato colonized by *R. irregularis* and *G. margarita* at five wpi. AMF, non-colonized tomato roots; *R. irregularis, R. irregularis*-colonized roots; *G. margarita*, *G. margarita*-colonized roots. The bars and dots indicate the average and individual values, respectively. The error bars indicate the standard errors (n = 9). The different letters indicate statistical significance (*p* < 0.05) in analysis of variance (ANOVA) with a post hoc Tukey-Kramer test.

Next, we compared the root colonization levels of the two AM fungi (Figure 2a). The quantification of AM fungal colonization revealed that the colonization rates of *G. margarita* were slightly lower than those of *R. irregularis*, although there was no statistical significance in Welch’s *t*-test. In addition, the fresh weight of the tomato shoots was significantly increased by the colonization of both *R. irregularis* and *G. margarita* at five weeks post-inoculation (wpi) (Figure 2b). Taken together, the symbiotic phenotypes were comparable to each other, except for the AM morphotype. 

### 2.2. Comparative Transcriptomics of Tomato Mycorrhizal Roots Accommodating Different AM Fungi

**Figure 3:**
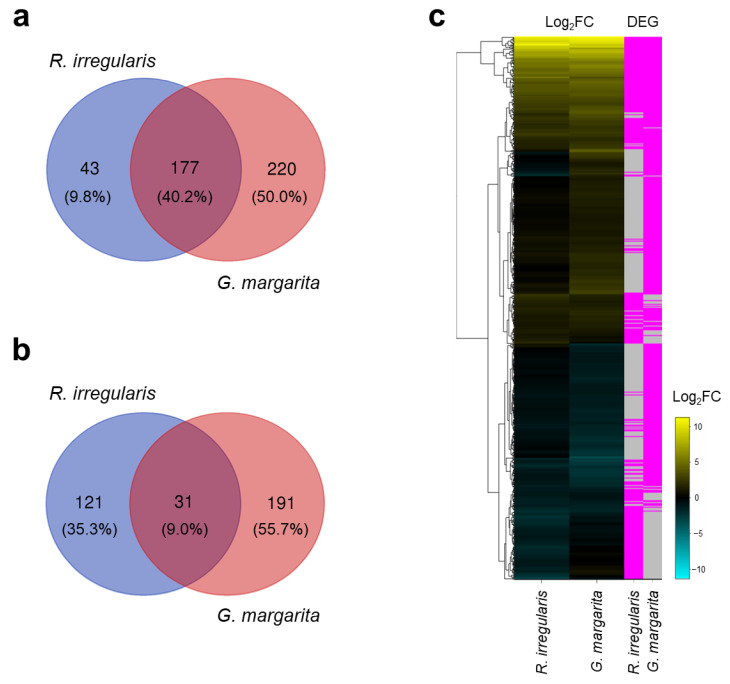
Differential expression analysis of tomato roots colonized by *Rhizophagus irregularis* or *Gigaspora margarita*. (**a**,**b**) Venn diagram of 440 AM-upregulated (**a**) and 343 AM-suppressed (**b**) genes compared with non-colonized roots at five weeks post-inoculation (wpi). Genes with |Log_2_Fold Change (FC)| > 1 and a false discovery rate (FDR) < 0.05 were considered differentially expressed genes (DEGs). (**c**) Hierarchical clustering of the total AM-responsive DEGs. The left-hand heatmap shows the Log_2_FC of genes expressed in colonized tomato roots relative to non-colonized roots. Blue indicates negative values; yellow, positive values; and black, zero change. The right-hand heatmap illustrates FDR values less than 0.05, with significant DEGs presented in pink. Detailed information about the DEGs can be found in Table S2.

Next, we conducted comparative transcriptomics between tomato roots separately colonized by *R. irregularis* and *G. margarita* to elucidate the regulatory mechanisms underlying *Arum*- and *Paris*-type AM symbioses. Our RNA-sequencing resulted in a minimum of 13 million and a maximum of 20 million raw sequence reads after trimming (Table S1). More than 80% of the filtered reads were uniquely mapped against the reference genome sequence of tomato [40]. The numbers of upregulated and downregulated differentially expressed genes (DEGs) in tomato mycorrhizae relative to non-colonized roots were 440 and 343, respectively (Figure 3a,b, Table S2). A comparison of DEGs between *R. irregularis*- and *G. margarita*-infected root samples revealed that the expression levels of 40.2% of genes were commonly promoted in both AM fungal colonizations compared with non-colonized roots (Figure 3a,c). In addition, 9.8% and 50.0% of the AM-upregulated DEGs were specific to *R. irregularis* and *G. margarita*, respectively (Figure 3a,c). On the contrary, AM-suppressed DEGs were primarily different between *R. irregularis*- and *G. margarita*-infected tomato roots (Figure 3b,c). 

**Figure 4:**
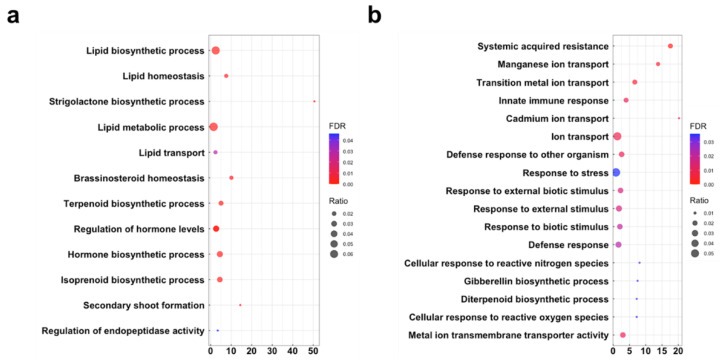
Gene ontology (GO) enrichment analysis on AM-upregulated genes. (**a**) GO terms enriched in the upregulated DEGs shared between tomato roots colonized by *Rhizophagus irregularis* or *Gigaspora margarita*. (**b**) GO terms enriched in DEGs upregulated only in roots infected by *G. margarita.* The representative GO terms obtained from the tested DEGs are listed. Bigger circles in the hierarchical clustering trees represent more genes annotated to the respective GO terms. The right-hand color bars indicate *p* values (< 0.05). Detailed information is presented in Table S3.

Gene ontology (GO) enrichment analysis was conducted to gain insight into how tomato plants respond to the associating AM fungi (Figure 4, Table S3). Since half of AM-upregulated DEGs was shared between *R. irregularis*- and *G. margarita*-colonized roots, we first examined the enriched GO terms in the common DEGs. We found that some GO terms related to the lipid biosynthesis and transport, and SL production was significantly enriched during association with both AM fungi (Figure 4a). On the contrary, AM-upregulated DEGs specific to *G. margarita* had enriched GO terms related to immune response, such as the response to biotic stimuli, systemic acquired resistance, and cellular response to reactive oxygen (Figure 4b). Interestingly, GA biosynthesis in tomato roots was also transcriptionally upregulated upon *G. margarita* infection. Since the number of DEGs unique to *R. irregularis* was not sufficient for enrichment analysis, the GO terms were not analyzed. 

We also conducted GO enrichment analysis for AM-suppressed DEGs (Figure 3b, Table S3). Among the shared DEGs downregulated in tomato roots colonized by *R. irregularis* or *G. margarita*, some GO terms corresponding to defense-related functions were again observed with statistical significance (Table S3). In addition, the GO terms associated with response to chitin and the salicylic acid biosynthetic process were significantly and uniquely detected in *R. irregularis*- and *G. margarita*-specific downregulated DEGs, respectively (Table S3).

### 2.3. Expression Patterns of AM-, SL-, and Defense-Related Genes in Tomato Roots against Different AM Fungal Colonizations

**Figure 5:**
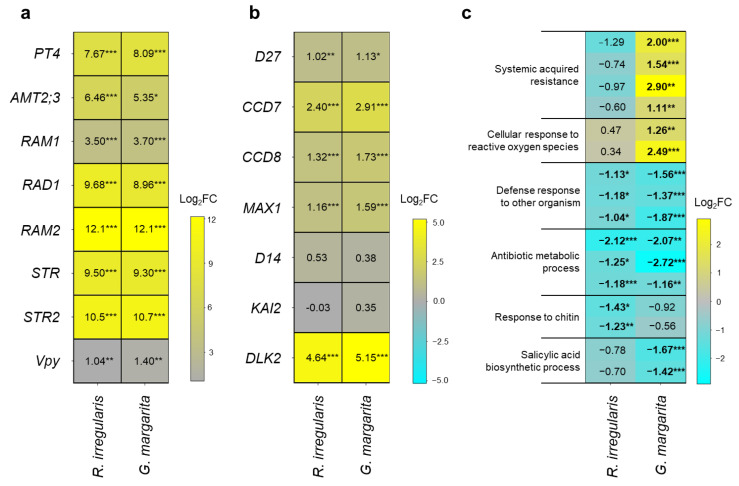
Expression patterns of AM-, strigolactone (SL)-, and defense-related genes during AM symbioses established by different AM fungi in tomato roots. At five weeks post-inoculation (wpi), the transcriptional response of the selected tomato genes against *Rhizophagus irregularis* or *Gigaspora margarita* colonization was analyzed. Log_2_-transformed fold changes (FC) of AM-related (**a**) and SL-related (**b**) genes versus the non-colonized roots are presented in each left-hand heatmap. (**c**) The expression patterns of DEGs involved in the defense-related pathways (Figure 4b, Table S3). The calculated values are written on the heatmaps. The Log_2_FC values of DEGs are shown in bold. Asterisks indicate the significance in the Log_2_FC (*: false discovery rate (FDR) < 0.05, **: FDR < 0.01, and ***: FDR < 0.001). The Log_2_FC and FDR values are listed in Table S4.

Several GO terms corresponding to membrane transport, lipid biosynthesis, and SL production were significantly enriched in the shared AM-upregulated DEGs. Therefore, we studied the expression pattern of AM- and SL-related genes upon different AM fungal colonizations. For this analysis, we selected *S. lycopersicum PT4* (*SlPT4*), *SlAMT2;3*, *SlRAM1*, *SlRAD1*, *SlRAM2*, *SlSTR*/*SlSTR2*, and *SlVpy* (Table S4). AM fungal colonization significantly upregulated the transcription of all the selected genes irrespective of the associating fungal species (Figure 5a, Table S4). These results were in line with our GO enrichment analysis.

Next, we analyzed the expression patterns of SL-related genes upon AM fungal colonization. For the analysis, we selected *SlD27*, *SlCCD7*, *SlCCD8*, and *SlMAX1* for the SL biosynthetic process and *SlD14*, *SlKAI2*, and *SlDLK2* for SL perception and signaling (Table S4) [41,42]. The expression levels of *SlD14* and *SlKAI2* were comparable to levels in non-colonized tomato roots (Figure 5b, Table S4). On the contrary, all of the selected SL biosynthetic genes and *SlDLK2* were transcriptionally upregulated by *R. irregularis* or *G. margarita* infection relative to the non-colonized roots (Figure 5b, Table S4). Together, these expression patterns of selected genes were consistent with the results of the GO enrichment analysis on the common AM-upregulated DEGs (Figure 4). 

Since our GO enrichment analysis also revealed transcriptional changes in defense-related pathways (Figure 4b, Table S4), the expression patterns of the corresponding genes were investigated. We determined that genes functioning in “systemic acquired resistance” and “cellular response to reactive oxygen species (ROS)” were specifically expressed upon *G. margarita* infection (Figure 5c). In addition, the gene expression patterns associated with “response to chitin” and “salicylic acid biosynthetic process” were consistent with the GO enrichment analysis (Figure 5c, Table S4). On the contrary, the expression levels of some genes involved in “defense response to other organism” and “antibiotic metabolic process” were commonly decreased by each AM fungal colonization (Figure 5c, Table S4). These results indicate that *R. irregularis* suppressed defense responses in tomato roots, whereas *G. margarita* also stimulated some parts of the immune pathways.

## 3. Discussion

AM morphotypes are known to change depending mainly on host traits. In previous studies, we used several host species and one AM fungal species to compare the molecular mechanisms underlying AM symbioses forming different morphotypes [31,32]. In this study, we focused on the transcriptional responses occurring in a single host species, tomato, associating with two AM fungi, namely, *R. irregularis* and *G. margarita*. Our findings revealed some shared and different transcriptional responses in tomato roots when the associating fungi and resulting AM morphotypes are distinct. 

### 3.1. Shared Transcriptional Reprogramming upon Different AM Fungal Colonizations

This study found some shared transcriptional programs during AM symbioses in tomato roots colonized with *R. irregularis* or *G. margarita*. Membrane transport and lipid biosynthesis were transcriptionally activated in mycorrhizae accommodating *R. irregularis* or *G. margarita* (Figure 4a, Table S3). These results indicate that the nutrient exchange between the host plants and fungal symbionts is activated, which contributes to symbiotic growth promotion in tomato plants (Figure 2b). In line with these findings, the expression levels of several symbiosis-related genes required for phosphate transport (*SlPT4*), ammonium transport (*SlAMT2;3*), lipid production and transport (*SlRAM2*, *SlSTR*, *SlSTR2*), and *GRAS* transcription factors regulating these symbiotic genes (*SlRAM1*, *SlRAD1*) were commonly and significantly increased by both AM fungal colonizations (Figure 5a, Table S4). These findings indicate that the symbiotic exchange of nutrients between tomatoes and AM fungi is irrespective of AM morphotype. As for *SlVpy*, both *R. irregularis* and *G. margarita* infections enhanced the expression levels of *SlVpy*. This result indicates that *SlVpy* is necessary for the development of arbuscule in both *Arum*- and *Paris*-type AMs. 

Our transcriptome analysis also revealed another shared transcriptional reprogramming upon AM fungal colonization, the SL biosynthetic process. *R. irregularis* and *G. margarita* colonization transcriptionally upregulated all selected genes for SL production at five wpi (Figure 5b, Table S4). These results were consistent with our GO enrichment analysis (Figure 4a). Moreover, the expression of *SlDLK2* was also upregulated by the two AM fungi (Figure 5b, Table S4). Recent work has elucidated the involvement of *SlDLK2* in the negative regulation of arbuscule branching [43]. In addition to AM-upregulated *SlVpy* expression, the mechanisms underlying arbuscule formation would be shared in *Arum*- and *Paris*-type AMs.

### 3.2. Specific Responses to Paris-Type AM Symbiosis

With respect to the different responses of tomato to AM fungal species, some genes involved in biotic stimuli such as the defense response to a pathogen and ROS were transcriptionally upregulated during *Paris*-type AM symbiosis established by *G. margarita* (Figure 4b, Table S3). In general, the perception of pathogen-associated molecular patterns, such as chitin, by pattern recognition receptors (PRRs) and penetration of the plant cell wall, triggers ROS production and transcriptional activation of some defense-responsive genes [44,45]. In addition, the disruption of the plant cell wall by pathogen penetration releases oligomeric fragments of plant cell wall polysaccharides, referred to as damage-associated molecular patterns (DAMPs), and can cause PRR-mediated local defense responses [46]. Taken together, the continuous invasion of *G. margarita* hyphae into tomato cortical cells in *Paris*-type AM roots might promote ROS production by increasing DAMPs.

Interestingly, a previous study has demonstrated that colonization by Gigasporaceae fungi in tomato (*S. lycopersicum* cv. 76R) roots results in high but transient expression levels of some defense-related genes, such as *PATHOGENESIS*-*RELATED PROTEIN* *1* (*PR-1*) and extracellular acidic chitinase (*CHI3*) [6]. On the contrary, Gao et al. (2004) showed that relatively weak accumulations of defense-related genes are observed in tomato roots forming *Arum*-type AM with the Glomeraceae family, such as *R. irregularis* and *Glomus mosseae*. Therefore, the continuous invasion of intracellular hyphae of *G. margarita* forming *Paris*-type AM roots possibly activates biotic responses in tomato roots. In fact, we found that *G. margarita* colonization significantly upregulated some defense pathways in tomato roots (Figure 4b and Figure 5c, Tables S3 and S4).

Nevertheless, our study demonstrated that *G. margarita* could colonize tomato roots comparably to *R. irregularis* (Figure 2a). Recent studies have shown that LCOs, CO4, and several small, secreted proteins derived from AM fungi alleviate the immune responses in host plants [3,5,9,47], which are consistent with the AM-suppressed chitin response and salicylic acid biosynthesis (Figure 5c, Table S3). These findings lead us to predict that AM fungal chitin oligomers and/or effectors might enable the two fungi to effectively colonize tomato plants by compromising defense responses, which might show comparable fungal colonization in tomato roots (Figure 2a). In addition, the function of SAR- and ROS-related genes whose expression levels were increased by *G. margarita* infection might be irrelevant or insufficient to inhibit fungal colonization. These ideas need further investigation.

Some GO terms associated with manganese or divalent metal transport were enriched within DEGs in *G. margarita*-colonized roots (Figure 4b, Table S3). One of the annotated genes, Solyc02g092800.3, is known as *Natural Resistance-Associated Macrophage Protein 1* (*NAMP*) metal transporter [48]. The *NRAMP* genes in legume plants have been reported to be transcriptionally upregulated in root nodules and localized on the peribacteroid membrane, where host plants and rhizobia exchange nutrients [49,50]. Therefore, tomato plants could take up divalent metals, such as iron and manganese, from intraradical *G. margarita* hyphae and utilize them for their growth.

### 3.3. Transcriptional Activation of the GA Biosynthetic Process

Our previous work demonstrated that bioactive GA production is upregulated in *E. grandiflorum Paris*-type AMs [31]. AM-promoted bioactive GA accumulation has also been reported in *Lotus japonicus* forming *Arum*-type AM roots [34]. However, our GO enrichment analysis revealed transcriptional upregulation of the GA biosynthetic process only in tomato roots forming *Paris*-type AM with *G. margarita* (Figure 4b, Table S3). In addition, we found that *Paris*-type AM symbiosis in *E. grandiflorum* and *Primula malacoides* is promoted by exogenous GA treatment [31]. Taken together, bioactive GAs might contribute to the establishment of *Paris*-type AM symbiosis in tomatoes colonized by *G. margarita*; however, this hypothesis needs to be investigated further.

## 4. Materials and Methods

### 4.1. Biological Materials and Growth Conditions

Seeds of *Solanum lycopersicum* L. cv. Micro-Tom were obtained from the University of Tsukuba, Tsukuba Plant Innovation Research Center, through the National Bio-Resource Project. The seeds were cleaned with 70% ethanol and then rinsed twice with sterilized distilled water. The pre-washed seeds were immersed and agitated in 1.5% (*v*/*v*) NaClO solution for 15 min. After the solution was removed, the seeds were set on two pieces of filter paper in a light chamber at 24 °C and a 14-h light/10-h dark/light cycle and incubated for six days. Spores of *Gigaspora margarita* MAFF520052 were obtained from the Genebank Project (National Agriculture and Food Research Organization, Japan) and sterilized in 0.1% (*v*/*v*) NaClO and 0.04% (*v*/*v*) Tween-20 for 15 min, followed by replacement of the solution with sterilized distilled water. *Rhizophagus irregularis* DAOM197198 spores were purchased from Premier Tech (Quebec, Canada). 

For the inoculation of six-day-old tomato seedlings with *R. irregularis*, 50 mL of 1/5 strength Hoagland solution (20-µM inorganic phosphate) containing 3000 spores was poured into a washed and autoclaved (121 °C for 20 min) 300-mL soil mixture of river sand and shibanome soil (2:1, *v*:*v*). Three tomato seedlings were then transplanted to the soil mixture. Each six-day-old tomato seedling was directly inoculated with 20 *G. margarita* spores. The inoculated seedlings were grown under the same conditions for five weeks. Subsequently, the lateral roots were harvested, and the fresh weight of shoots was measured.

### 4.2. Observation and Quantification of Mycorrhizal Roots

AM fungal colonization was quantified by staining the root samples with trypan blue and microscopically observing them as previously reported [31]. Briefly, mycorrhizal roots were fixed in a FAA solution (5% formaldehyde, 5% acetic acid, and 45% ethanol [*v*/*v*]), rinsed twice with distilled water. The fixed root samples were heated at 90 °C for 15 min and neutralized in 2% HCl solution. Subsequently, the root samples were immersed in trypan blue diluted by lactic acid at 0.05% and heated at 90 °C for 15 min. The stained samples were sliced with a scalpel under a SZX16 stereomicroscope (Olympus, Tokyo, Japan), and images were taken using a BX53 light microscope (Olympus) equipped with a digital camera (DP27; Olympus).

### 4.3. Extraction of RNA from Tomato Roots

To conduct transcriptome analysis by RNA-sequencing (RNA-seq), we prepared RNA samples from fresh tomato roots. The lateral roots from three seedlings in a nuclease-free tube (INA-OPTIKA, Osaka, Japan) containing two 5-mm beads were frozen in liquid nitrogen and then homogenized in a ShakeMan6 (Bio-Medical Science, Tokyo, Japan); afterward, 450 μL of Fruit-mate for RNA Purification (Takara Bio, Shiga, Japan) was added to each tube. After thoroughly mixing the tubes, the slurry was transferred to another tube and centrifuged at 12,000× *g* at 4 °C for 5 min. The supernatant was mixed with 450-μL ethanol and RNAiso Plus (Takara) in a new tube. The genomic DNA-free total RNA was prepared using a Zymo-spin IIICG Column (Zymo Research, Orange, CA, USA). The column membrane was treated with DNaseI (Takara) before extracting the RNA according to the manufacturer’s protocols. The purity and quantity of total RNA were measured at 260 and 280 nm (*A*260: *A*280) using DeNovix DS-11 + (Scrum, Tokyo, Japan). The prepared samples were stored at −80 °C until use.

### 4.4. RNA-seq, Data Analysis, and Gene Identification

The library preparation and RNA-seq were performed by Genewiz (Tokyo, Japan) using DNBSEQ-G400 and resulted in more than 14 million strand-specific paired-end (2 × 150 bp) reads per sample (Table S1). The obtained raw reads were filtered (<QV30), and the adapter sequence was trimmed using Fastp [51]. The purified single-end reads were mapped to tomato genome sequence version SL4.0 and annotation ITAG4.0 built by the International Tomato Genome Sequencing Project (https://solgenomics.net/organism/Solanum_lycopersicum/genome, accessed on 2 December 2021) using the STAR program [52]. The resulting data were processed with featureCounts v1.6.4 [53] to quantify gene expression. Then, we extracted the DEGs using the EdgeR package [54] in the R software v4.0.2. In this study, genes with FDR < 0.05 were considered DEGs. In addition, the GO terms significantly enriched within the DEG datasets were identified using Shiny GO v0.61 [55]. Tomato genes with zero count in at least one of the root samples were removed before expression pattern and GO enrichment analyses. To investigate the effects of *R. irregularis* and *G. margarita* infections on the expression of AM symbiosis- or SL-related genes, we selected several tomato genes required for the mutualism. To this end, known genes in *M. truncatula* and *Arabidopsis thaliana* were used as queries for tBLASTx searches in the Sol Genomics Network using the default setting (Table S4). Raw nucleotide sequence data from this study are available from the DDBJ Sequence Read Archive under accession number DRA013369.

### 4.5. Biological Replicates, Statistical Analysis, and Heatmap Production

To quantify the root colonization rate (%), we considered ten pieces of root fragment from one tomato sample on a microscope slide as one biological replicate. When preparing a sample for RNA-seq, one pool of total RNA extracted from three seedlings was one biological replicate. Finally, we used three RNA samples for the RNA-seq. Statistical analysis was conducted in the R software v4.0.2. Welch’s *t*-test and analysis of variance (ANOVA) followed by a post hoc Tukey-Kramer test were applied for the colonization rates and shoot fresh weight, respectively. The R package heatmaply was used to draw the heatmaps [56].

## 5. Conclusions

We found some common AM-specific transcriptional programs for the membrane transport, arbuscule development, and SL biosynthetic pathway in *Arum*- and *Paris*-type AM symbioses in tomato roots. These similarities indicate that the primary and downstream mechanisms for accommodating AM fungi would be common, irrespective of the AM morphotype. In addition, our study demonstrated that the colonization of phylogenetically distant AM fungi differentially affected the defense-related pathways and GA biosynthetic process in tomato roots. These different transcriptional responses would enable tomatoes to fine-tune the mutualism between tomato roots and diverse AM fungi to optimize the host growth. In addition, the upstream regulation underlying *Arum*- and *Paris*-type AM symbioses in tomato plants might be differentially modulated by GA. Further investigation of how GA regulates *Paris*-type AM symbiosis established by tomato roots and *G. margarita* would improve our understanding of the regulatory mechanisms underpinning AM symbioses.

## Data Availability

The nucleotide sequence data obtained from our RNA sequencing has been deposited into the DDBJ Sequence Read Archive under the accession number DRA013369.

## Supplementary Materials

The following supporting information can be downloaded at: https://www.mdpi.com/article/10.3390/plants11060747/s1, Table S1: Results of RNA-sequencing, read mapping, and read count; Table S2: Information of symbiosis-responsive DEGs; Table S3: Gene ontology enrichment analysis on tomato mycorrhizae; Table S4: Blastp results and expression patterns of genes listed in Figure 5.
